# Pseudo almost periodic solutions for quaternion-valued cellular neural networks with discrete and distributed delays

**DOI:** 10.1186/s13660-018-1837-1

**Published:** 2018-09-19

**Authors:** Xiaofang Meng, Yongkun Li

**Affiliations:** Department of Mathematics, Yunnan University, Kunming, People’s Republic of China

**Keywords:** 34K14, 34K20, 92B20, Quaternion-valued cellular neural networks, Distributed delay, Pseudo almost periodic solutions, Exponent stability

## Abstract

This paper is concerned with a class of quaternion-valued cellular neural networks with discrete and distributed delays. By using the exponential dichotomy of linear systems and a fixed point theorem, sufficient conditions are derived for the existence and global exponential stability of pseudo almost periodic solutions of this class of neural networks. Finally, a numerical example is given to illustrate the feasibility of the obtained results.

## Introduction

Since Chua and Yang proposed cellular neural networks (CNNs) in 1988 [1], various dynamical behaviors of CNNs, such as the existence and stability of the equilibrium, periodic solutions, anti-periodic solutions, almost periodic solutions, and pseudo-almost periodic solutions, have been studied by many scholars [2–15].

On the one hand, quaternion-valued neural networks (QVNNs), as an extension of the complex-valued neural networks (CVNNs), can deal with multi-level information and require only half the connection weight parameters of CVNNs [16]. Moreover, compared with CVNNs, QVNNs perform more prominently when it comes to geometrical transformations, like 2D affine transformations or 3D affine transformations. 3D geometric affine transformations can be represented efficiently and compactly based on QVNNs, especially spatial rotation [17]. Since the multiplication of quaternion is not commutative due to Hamilton rules: $ij=-ji=k$, $jk=-kj=i, ki=-ik=j, i^{2}=j^{2}=k^{2}=ijk=-1$, the analysis for QVCNNs becomes difficult. However, with the continuous development of the theory of quaternion, there are some results about the dynamics of QVNNs. For example, the authors of [18, 19] studied the existence and global exponential stability of equilibrium point for QVNNs; the authors of [20] investigated the robust stability of QVNNs with time delays and parameter uncertainties; the authors of [21] considered the existence and stability of pseudo almost periodic solutions for a class of QVCNNs on time scales by a special decomposition method; the authors of [22, 23] investigated the existence and global *μ*-stability of an equilibrium point for QVNNs; the authors of [24] dealt with the existence and stability of periodic solutions for QVCNNs by using a continuation theorem of coincidence degree theory; the authors of [25] studied the almost periodic synchronization for QVCNNs. Although non-autonomous neural networks are more general and practical than the autonomous ones, up to now, there have been only few results about the dynamic behaviors of non-autonomous QVNNs.

On the other hand, it is well known that the periodicity, almost periodicity, pseudo almost periodicity, and so on are the very important dynamics for non-autonomous systems [10, 12, 26]. Moreover, the almost periodicity is more general than the periodicity. In addition, the pseudo almost periodicity is a natural generalization of almost periodicity. In the past few years, the pseudo almost periodicity of real-valued neural networks (RVNNs) has been studied by many authors [13–15, 27–34]. Besides, as we all know, time delay is universal and can change the dynamical behavior of the system under consideration [3, 5, 29, 30, 35, 36]. Therefore, it is important and necessary to consider the neural network model with time delay. However, to the best of our knowledge, there is no paper published on the existence and stability of pseudo almost periodic solutions for quaternion-valued cellular neural networks (QVCNNs) with discrete and distributed delays.

Motivated by the above, in this paper, we are concerned with the following QVCNN with discrete and distributed delays:

1 $$\begin{aligned} x_{p}'(t) =&-c_{p}(t)x_{p}(t)+ \sum_{q=1}^{n}a_{pq}(t)f_{q} \bigl(x_{q} \bigl(t-\tau _{pq}(t) \bigr) \bigr) \\ &+\sum_{q=1}^{n}b_{pq}(t) \int _{0}^{\infty }K_{pq}(u)g_{q} \bigl(x_{q}(t-u) \bigr)\,\mathrm{d}u+u_{p}(t), \end{aligned}$$

where $p\in \{1,2,\ldots ,n\}:=\Lambda $, $x_{p}(t)\in \mathbb{Q}$ is the state vector of the *p*th unit at time *t*, $c_{p}(t)>0$ represents the rate at which the *p*th unit will reset its potential to the resting state in isolation when disconnected from the network and external inputs, $a_{pq}(t), b_{pq}(t)\in \mathbb{Q}$ are the synaptic weights of delayed feedback between the *p*th neuron and the *q*th neuron, $f_{q}, g_{q}:\mathbb{Q}\rightarrow \mathbb{Q}$ are the activation functions of signal transmission, $\tau _{pq}(t)\geq 0$ denotes the transmission delay, $u_{p}(t)\in \mathbb{Q}$ denotes the external input on the *p*th neuron at time *t*.

Throughout this paper, we denote by $BC(\mathbb{R},\mathbb{R}^{n})$, the set of all bounded continuous functions from $\mathbb{R}$ to $\mathbb{R}^{n}$.

The initial value is given by

$$\begin{aligned} x_{p}(s)=\phi _{p}(s),\quad s\in (-\infty ,0], p\in \Lambda , \end{aligned}$$

where $\phi _{p}\in BC((-\infty ,0],\mathbb{Q})$.

Our main aim in this paper is to study the existence and global exponential stability of pseudo almost periodic solutions of (1). The main contributions of this paper are listed as follows.

1. To the best of our knowledge, this is the first time to study the existence and stability of pseudo almost periodic solutions for QVCNNs with discrete and distributed delays.
2. The stability of QVNNs with distributed delays has not been reported yet. Therefore, our result about the stability of QVNNs is new, and most of the existing results about the stability of QVNNs are obtained by using the theory of linear matrix inequalities but ours are not.
3. The method that we use to transform QVNNs into RVNNs is different from that used in [18, 20–23].
4. QVCNN (1) contains RVCNNs and CVCNNs as its special cases.

Throughout this paper, $\mathbb{R}^{n\times n}$, $\mathbb{Q}^{n\times n}$ denote the set of all $n\times n$ real-valued and quaternion-valued matrices, respectively. The skew field of quaternion is denoted by

$$\begin{aligned} \mathbb{Q}:= \bigl\{ x=x^{R}+ix^{I}+jx^{J}+kx^{K} \bigr\} , \end{aligned}$$

where $x^{R}$, $x^{I}$, $x^{J}$, $x^{K}$ are real numbers and the elements *i*, *j*, and *k* obey Hamilton’s multiplication rules.

For the convenience, we will introduce the notations: $\bar{h}=\sup_{t\in \mathbb{R}}\vert h(t) \vert $, $\underline{h}=\inf_{t\in \mathbb{R}}\vert h(t) \vert $, where $h(t)$ is a bounded continuous function.

This paper is organized as follows. In Sect. 2, we introduce some definitions, make some preparations for later sections. In Sect. 3, by utilizing Banach’s fixed point theorem and differential inequality techniques, we establish the existence and global exponential stability of pseudo almost periodic solutions of (1). In Sect. 4, we give an example to demonstrate the feasibility of our results. This paper ends with a brief conclusion in Sect. 5.

## Preliminaries

In this section, we shall first recall some fundamental definitions, lemmas which are used in what follows.

### Definition 1

([37])

A function $u\in BC(\mathbb{R},\mathbb{R}^{n})$ is said to be almost periodic if, for any $\epsilon >0$, it is possible to find a real number $l=l(\epsilon )>0$, for any interval with length $l(\epsilon )$, there exists a number $\tau =\tau (\epsilon )$ in this interval such that $\vert u(t+\tau )-u(t) \vert <\epsilon $ for all $t\in \mathbb{R}$. The collection of such functions will be denoted by $AP(\mathbb{R},\mathbb{R}^{n})$.

Let

$$\begin{aligned} PAP_{0} \bigl(\mathbb{R},\mathbb{R}^{n} \bigr) = \biggl\{ f \in BC \bigl(\mathbb{R},\mathbb{R}^{n} \bigr) \Bigm| \lim _{r\rightarrow +\infty }\frac{1}{2r} \int _{-r}^{r}\bigl\Vert f(t) \bigr\Vert \, \mathrm{d}t=0 \biggr\} . \end{aligned}$$

### Definition 2

([38, 39])

A function $f\in BC(\mathbb{R},\mathbb{R}^{n})$ is called pseudo almost periodic if it can be expressed as $f=f_{1}+f_{0}$, where $f_{1}\in AP(\mathbb{R},\mathbb{R}^{n})$ and $f_{0}\in PAP_{0}(\mathbb{R},\mathbb{R}^{n})$. The collection of such functions will be denoted by $PAP(\mathbb{R},\mathbb{R}^{n})$.

From the above definitions, it is easy to see that $AP(\mathbb{R},\mathbb{R}^{n})\subset PAP(\mathbb{R},\mathbb{R}^{n})$.

### Definition 3

A quaternion-valued function $x=x^{R}+ix^{I}+jx^{J}+kx^{K}\in BC(\mathbb{R},\mathbb{Q}^{n})$ is called a pseudo almost periodic function if, for every $l\in \{R,I,J,K\}:=E$, $x^{l}\in PAP(\mathbb{R},\mathbb{R}^{n})$.

### Definition 4

([38, 39])

The system

2 $$\begin{aligned} x'(t)=A(t)x(t) \end{aligned}$$

is said to admit an exponential dichotomy if there exist a projection *P* and positive constants $\alpha ,\beta $ such that the fundamental solution matrix $X(t)$ satisfies

$$\begin{aligned} &\bigl\vert X(t)PX^{-1}(s) \bigr\vert \leq \beta e^{-\alpha (t-s)},\quad t\geq s, \\ &\bigl\vert X(t) (I-P)X^{-1}(s) \bigr\vert \leq \beta e^{-\alpha (s-t)},\quad t\leq s. \end{aligned}$$

Consider the following pseudo almost periodic system:

3 $$\begin{aligned} x'(t)=A(t)x(t)+f(t), \end{aligned}$$

where $A(t)$ is an almost periodic matrix function, $f(t)$ is a pseudo almost periodic vector function.

### Lemma 1

([38, 39])

*If the linear system* (2) *admits an exponential dichotomy*, *then system* (3) *has a unique pseudo almost periodic solution*:

$$ x(t)= \int _{-\infty }^{t}X(t)PX^{-1}(s)f(s)\, \mathrm{d} s- \int _{t}^{+\infty }X(t) (I-P)X^{-1}(s)f(s) \, \mathrm{d} s, $$

*where* $X(t)$ *is the fundamental solution matrix of* (2).

### Lemma 2

([38, 39])

*Let* $c_{p}(t)$ *be an almost periodic function on* $\mathbb{R}$ *and*

$$ M[c_{p}]=\lim_{T\rightarrow \infty }\frac{1}{T} \int _{t}^{t+T}c_{p}(s)\,\mathrm{d} s>0, \quad p\in \Lambda . $$

*Then the linear system*

$$\begin{aligned} x'(t)=\operatorname{diag} \bigl(-c_{1}(t),-c_{2}(t), \dots ,-c_{n}(t) \bigr)x(t) \end{aligned}$$

*admits an exponential dichotomy on* $\mathbb{R}$.

In order to decompose the quaternion-valued system (1) into a real-valued system, we need the following assumption:

$(S_{1})$
Let $x_{p}=x_{p}^{R}+ix_{p}^{I}+jx_{p}^{J}+kx_{p}^{K}$, $x_{p}^{l}\in \mathbb{R}, l\in E$. Then the activation functions $f_{q}(x_{q})$ and $g_{q}(x_{q})$ of (1) can be expressed as
  $$\begin{aligned}& f_{q}(x_{q}) = f_{q}^{R} \bigl(x_{q}^{R},x_{q}^{I},x_{q}^{J},x_{q}^{K} \bigr) \\& \hphantom{f_{q}(x_{q}) =}{}+if_{q}^{I} \bigl(x_{q}^{R},x_{q}^{I},x_{q}^{J},x_{q}^{K} \bigr)+jf_{q}^{J} \bigl(x_{q}^{R},x_{q}^{I},x_{q}^{J},x_{q}^{K} \bigr) +kf_{q}^{K} \bigl(x_{q}^{R},x_{q}^{I},x_{q}^{J},x_{q}^{K} \bigr), \\& g_{q}(x_{q}) = g_{q}^{R} \bigl(x_{q}^{R},x_{q}^{I},x_{q}^{J},x_{q}^{K} \bigr) +ig_{q}^{I} \bigl(x_{q}^{R},x_{q}^{I},x_{q}^{J},x_{q}^{K} \bigr)+jg_{q}^{J} \bigl(x_{q}^{R},x_{q}^{I},x_{q}^{J},x_{q}^{K} \bigr) \\& \hphantom{f_{q}(x_{q}) =}{} +kg_{q}^{K} \bigl(x_{q}^{R},x_{q}^{I},x_{q}^{J},x_{q}^{K} \bigr), \end{aligned}$$
  where $f_{q}^{l}, g_{q}^{l}:\mathbb{R}^{4}\rightarrow \mathbb{R}$, $p\in \Lambda , l\in E$.

Under assumption $(S_{1})$, system (1) can be decomposed into the following four real-valued sub-systems:

4 $$\begin{aligned}& \bigl(x_{p}^{R}(t) \bigr)' =-c_{p}(t)x_{p}^{R}(t)+ \sum _{q=1}^{n} \bigl(a_{pq}^{R}(t)f_{q}^{R}[t,x] -a_{pq}^{I}(t)f_{q}^{I}[t,x] \\ & \hphantom{ (x_{p}^{R}(t) )' =}{}-a_{pq}^{J}(t)f_{q}^{J}[t,x] -a_{pq}^{K}(t)f_{q}^{K}[t,x] \bigr)+\sum _{q=1}^{n} \biggl(b_{pq}^{R}(t) \int _{0}^{\infty }K_{pq}(u) \\ & \hphantom{ (x_{p}^{R}(t) )' =}{}\times g_{q}^{R}[t,u,x]\,\mathrm{d}u -b_{pq}^{I}(t) \int _{0}^{\infty }K_{pq}(u)g_{q}^{I}[t,u,x] \,\mathrm{d}u \\ & \hphantom{ (x_{p}^{R}(t) )' =}{}-b_{pq}^{J}(t) \int _{0}^{\infty }K_{pq}(u)g_{q}^{J}[t,u,x] \,\mathrm{d}u -b_{pq}^{K}(t) \int _{0}^{\infty }K_{pq}(u) \\ & \hphantom{ (x_{p}^{R}(t) )' =}{}\times g_{q}^{K}[t,u,x]\,\mathrm{d}u \biggr)+u_{p}^{R}(t), \end{aligned}$$

5 $$\begin{aligned}& \bigl(x_{p}^{I}(t) \bigr)' = -c_{p}(t)x_{p}^{I}(t)+ \sum _{q=1}^{n} \bigl(a_{pq}^{R}(t)f_{q}^{I}[t,x] +a_{pq}^{I}(t)f_{q}^{R}[t,x] \\ & \hphantom{ (x_{p}^{I}(t) )'=}{} +a_{pq}^{J}(t)f_{q}^{K}[t,x] -a_{pq}^{K}(t)f_{q}^{J}[t,x] \bigr)+\sum _{q=1}^{n} \biggl(b_{pq}^{R}(t) \int _{0}^{\infty }K_{pq}(u) \\ & \hphantom{ (x_{p}^{I}(t) )'=}{} \times g_{q}^{I}[t,u,x]\,\mathrm{d}u +b_{pq}^{I}(t) \int _{0}^{\infty }K_{pq}(u)g_{q}^{R}[t,u,x] \,\mathrm{d}u \\ & \hphantom{ (x_{p}^{I}(t) )'=}{} +b_{pq}^{J}(t) \int _{0}^{\infty }K_{pq}(u)g_{q}^{K}[t,u,x] \,\mathrm{d}u -b_{pq}^{K}(t) \int _{0}^{\infty }K_{pq}(u) \\ & \hphantom{ (x_{p}^{I}(t) )'=}{} \times g_{q}^{J}[t,u,x]\,\mathrm{d}u \biggr)+u_{p}^{I}(t), \end{aligned}$$

6 $$\begin{aligned}& \bigl(x_{p}^{J}(t) \bigr)' = -c_{p}(t)x_{p}^{J}(t)+ \sum _{q=1}^{n} \bigl(a_{pq}^{R}(t)f_{q}^{J}[t,x] +a_{pq}^{J}(t)f_{q}^{R}[t,x] \\ & \hphantom{ (x_{p}^{I}(t) )'=}{} -a_{pq}^{I}(t)f_{q}^{K}[t,x] +a_{pq}^{K}(t)f_{q}^{I}[t,x] \bigr)+\sum _{q=1}^{n} \biggl(b_{pq}^{R}(t) \int _{0}^{\infty }K_{pq}(u) \\ & \hphantom{ (x_{p}^{I}(t) )'=}{} \times g_{q}^{J}[t,u,x]\,\mathrm{d}u +b_{pq}^{J}(t) \int _{0}^{\infty }K_{pq}(u)g_{q}^{R}[t,u,x] \,\mathrm{d}u \\ & \hphantom{ (x_{p}^{I}(t) )'=}{} -b_{pq}^{I}(t) \int _{0}^{\infty }K_{pq}(u)g_{q}^{K}[t,u,x] \,\mathrm{d}u +b_{pq}^{K}(t) \int _{0}^{\infty }K_{pq}(u) \\ & \hphantom{ (x_{p}^{I}(t) )'=}{} \times g_{q}^{I}[t,u,x]\,\mathrm{d}u \biggr)+u_{p}^{J}(t), \end{aligned}$$

7 $$\begin{aligned}& \bigl(x_{p}^{K}(t) \bigr)' = -c_{p}(t)x_{p}^{K}(t)+ \sum _{q=1}^{n} \bigl(a_{pq}^{R}(t)f_{q}^{K}[t,x] +a_{pq}^{K}(t)f_{q}^{R}[t,x] \\ & \hphantom{ (x_{p}^{I}(t) )'=}{} +a_{pq}^{I}(t)f_{q}^{J}[t,x] -a_{pq}^{J}(t)f_{q}^{I}[t,x] \bigr)+\sum _{q=1}^{n} \biggl(b_{pq}^{R}(t) \int _{0}^{\infty }K_{pq}(u) \\ & \hphantom{ (x_{p}^{I}(t) )'=}{} \times g_{q}^{K}[t,u,x]\,\mathrm{d}u +b_{pq}^{K}(t) \int _{0}^{\infty }K_{pq}(u)g_{q}^{R}[t,u,x] \,\mathrm{d}u \\ & \hphantom{ (x_{p}^{I}(t) )'=}{} +b_{pq}^{I}(t) \int _{0}^{\infty }K_{pq}(u)g_{q}^{J}[t,u,x] \,\mathrm{d}u -b_{pq}^{J}(t) \int _{0}^{\infty }K_{pq}(u) \\& \hphantom{ (x_{p}^{I}(t) )'=}{} \times g_{q}^{I}[t,u,x]\,\mathrm{d}u \biggr)+u_{p}^{K}(t), \end{aligned}$$

where $f_{q}^{l}[t,x]\triangleq f_{q}^{l} (x_{q}^{R}(t-\tau _{pq}(t)),x_{q}^{I}(t-\tau _{pq}(t)),x_{q}^{J}(t-\tau _{pq}(t)), x_{q}^{K}(t-\tau _{pq}(t)) )$, $g_{q}^{l}[t,u,x]\triangleq g_{q}^{l} (x_{q}^{R}(t-u),(x_{q}^{I}(t-u)),(x_{q}^{J}(t-u)),(x_{q}^{K}(t-u)) )$, and

$$\begin{aligned}& a_{pq}(t) = a_{pq}^{R}(t)+ia_{pq}^{I}(t)+ja_{pq}^{J}(t)+ka_{pq}^{K}(t), \\& b_{pq}(t) = b_{pq}^{R}(t)+ib_{pq}^{I}(t)+jb_{pq}^{J}(t)+kb_{pq}^{K}(t), \\& u_{p}(t) = u_{p}^{R}(t)+iu_{p}^{I}(t)+ju_{p}^{J}(t)+ku_{p}^{K}(t). \end{aligned}$$

According to (4)–(7), one can obtain that

8 $$\begin{aligned} X_{p}'(t) =&-c_{p}(t)X_{p}(t)+ \sum_{q=1}^{n}A_{pq}(t)F_{q}[t,x] \\ &+\sum_{q=1}^{n}B_{pq}(t) \int _{0}^{\infty }K_{pq}(u)G_{q}[t,u,x] \,\mathrm{d}u+U_{p}(t),\quad p\in \Lambda , \end{aligned}$$

where

$$\begin{aligned} A_{pq}(t)&= \left ( \textstyle\begin{array}{cccc} a_{pq}^{R}(t)&-a_{pq}^{I}(t)&-a_{pq}^{J}(t)&-a_{pq}^{K}(t) \\ a_{pq}^{I}(t)&a_{pq}^{R}(t)&-a_{pq}^{K}(t)&a_{pq}^{J}(t) \\ a_{pq}^{J}(t)&a_{pq}^{K}(t)&a_{pq}^{R}(t)&-a_{pq}^{I}(t) \\ a_{pq}^{K}(t)&-a_{pq}^{J}(t)&a_{pq}^{I}(t)&a_{pq}^{R}(t) \end{array}\displaystyle \right ) , \\ B_{pq}(t)&= \left ( \textstyle\begin{array}{cccc} b_{pq}^{R}(t)&-b_{pq}^{I}(t)&-b_{pq}^{J}(t)&-b_{pq}^{K}(t) \\ b_{pq}^{I}(t)&b_{pq}^{R}(t)&-b_{pq}^{K}(t)&b_{pq}^{J}(t) \\ b_{pq}^{J}(t)&b_{pq}^{K}(t)&b_{pq}^{R}(t)&-b_{pq}^{I}(t) \\ b_{pq}^{K}(t)&-b_{pq}^{J}(t)&b_{pq}^{I}(t)&b_{pq}^{R}(t) \end{array}\displaystyle \right ) ,\qquad X_{p}(t)= \left ( \textstyle\begin{array}{cccc} x_{p}^{R}(t)\\ x_{p}^{I}(t)\\ x_{p}^{J}(t) \\ x_{p}^{K}(t) \end{array}\displaystyle \right ) , \\ U_{p}(t) &= \left ( \textstyle\begin{array}{cccc} u_{p}^{R}(t)\\ u_{p}^{I}(t)\\ u_{p}^{J}(t) \\ u_{p}^{K}(t) \end{array}\displaystyle \right ) ,\qquad F_{q}[t,x]= \left ( \textstyle\begin{array}{cccc} f_{q}^{R}[t,x]\\ f_{q}^{I}[t,x]\\ f_{q}^{J}[t,x] \\ f_{q}^{K}[t,x] \end{array}\displaystyle \right ) ,\qquad G_{q}[t,u,x]= \left ( \textstyle\begin{array}{cccc} g_{q}^{R}[t,u,x]\\ g_{q}^{I}[t,u,x]\\ g_{q}^{J} [t,u,x]\\ g_{q}^{K}[t,u,x] \end{array}\displaystyle \right ) . \end{aligned}$$

The initial condition associated with (8) is of the form

$$\begin{aligned} X_{p}(s)=\Phi _{p}(s),\quad p\in \Lambda , s\in (-\infty ,0], \end{aligned}$$

where

$$\begin{aligned} \Phi _{p}(s)= \bigl( \phi _{p}^{R}(s), \phi _{p}^{I}(s), \phi _{p}^{J}(s), \phi _{p}^{K}(s) \bigr) ^{T} \end{aligned}$$

and $\phi _{p}^{l}(s)\in BC((-\infty ,0],\mathbb{R}), p\in \Lambda , l\in E$.

### Remark 1

Under assumption $(S_{1})$, it is easy to see that if $X=(x_{1}^{R},x_{1}^{I},x_{1}^{J},x_{1}^{K},\dots ,x_{n}^{R},x_{n}^{I}, x_{n}^{J}, x_{n}^{K})^{T}\in \mathbb{R}^{4n}$ is a solution of system (8), then $x=(X_{1},X_{2},\ldots ,X_{n})^{T}$ is a solution of system (1), and vice visa, where $X_{p}=x_{p}^{R}+ix_{p}^{I}+jx_{p}^{J}+kx_{p}^{K}, p\in \Lambda $. Therefore, to find a solution for system (1) is equivalent to finding one for system (8). To study the stability of solutions of system (1), we only need to investigate the stability of solutions of system (8).

## Main results

In this section, we establish the existence and global exponential stability of pseudo almost periodic solutions of system (8).

Let $\mathbb{X}=\{f(t)\mid f\in PAP(\mathbb{R},\mathbb{R}^{4n})\}$ with the norm $\Vert f \Vert _{\mathbb{X}}=\sup_{t\in \mathbb{R}}\Vert f(t) \Vert $, where $\Vert f(t) \Vert =\max_{1\leq h\leq 4n}\{\vert f_{h}(t) \vert \}$, then $\mathbb{X}$ is a Banach space.

In the following, we assume that the following conditions hold:

$(S_{2})$
There exist positive constants $\alpha _{q}^{l},\beta _{q}^{l}$ such that
  $$\begin{aligned}& \bigl\vert f_{q}^{l} \bigl(x_{q}^{R},x_{q}^{I},x_{q}^{J},x_{q}^{K} \bigr) -f_{q}^{l} \bigl(y_{q}^{R},y_{q}^{I},y_{q}^{J},y_{q}^{K} \bigr) \bigr\vert \\& \quad \leq \alpha _{q}^{R}\bigl\vert x_{q}^{R}-y_{q}^{R} \bigr\vert + \alpha _{q}^{I}\bigl\vert x_{q}^{I}-y_{q}^{I} \bigr\vert + \alpha _{q}^{J}\bigl\vert x_{q}^{J}-y_{q}^{J} \bigr\vert +\alpha _{q}^{K}\bigl\vert x_{q}^{K}-y_{q}^{K} \bigr\vert , \\& \bigl\vert g_{q}^{l} \bigl(x_{q}^{R},x_{q}^{I},x_{q}^{J},x_{q}^{K} \bigr) -g_{q}^{l} \bigl(y_{q}^{R},y_{q}^{I},y_{q}^{J},y_{q}^{K} \bigr) \bigr\vert \\& \quad \leq \beta _{q}^{R}\bigl\vert x_{q}^{R}-y_{q}^{R} \bigr\vert + \beta _{q}^{I}\bigl\vert x_{q}^{I}-y_{q}^{I} \bigr\vert + \beta _{q}^{J}\bigl\vert x_{q}^{J}-y_{q}^{J} \bigr\vert +\beta _{q}^{K}\bigl\vert x_{q}^{K}-y_{q}^{K} \bigr\vert \end{aligned}$$
  and $f_{q}^{l}(0,0,0,0)=g_{q}^{l}(0,0,0,0)=0$, where $p\in \Lambda $, $l\in E$.
$(S_{3})$
The function $c_{p}\in C(\mathbb{R},\mathbb{R}^{+})$ with $M[c_{p}]>0$ is almost periodic, $U_{p}\in C(\mathbb{R},\mathbb{R}^{4\times 1})$, $A_{pq},B_{pq}\in C(\mathbb{R},\mathbb{R}^{4\times 4})$, and $\tau _{pq}\in C(\mathbb{R},\mathbb{R}^{+})$ are pseudo almost periodic, where $p,q\in \Lambda $.
$(S_{4})$
The delay kernel $K_{pq}:[0,\infty )\rightarrow \mathbb{R}$ is continuous and integrable with $0\leq \int _{0}^{\infty } \vert K_{pq}(u) \vert \,\mathrm{d}u\leq \bar{K}_{pq}$, where $p,q\in \Lambda $.
$(S_{5})$
There exists a constant *κ* such that
  $$\begin{aligned} \max_{p\in \Lambda } \biggl\{ \max_{l\in E} \biggl\{ \frac{\Theta _{p}\kappa +\bar{u}_{p}^{l}}{\underline{c}_{p}} \biggr\} \biggr\} \leq \kappa ,\qquad \max_{p\in \Lambda } \biggl\{ \frac{\Theta _{p}}{\underline{c}_{p}} \biggr\} :=\rho < 1, \end{aligned}$$
  where
  $$\begin{aligned}& \Theta _{p} = V_{p}+W_{p},\quad p\in \Lambda , \\& V_{p} = \sum_{q=1}^{n} \bigl( \bar{a}_{pq}^{R}+\bar{a}_{pq}^{I}+ \bar{a}_{pq}^{J}+\bar{a}_{pq}^{K} \bigr) \bigl(\alpha _{q}^{R}+\alpha _{q}^{I}+ \alpha _{q}^{J}+\alpha _{q}^{K} \bigr), \quad p\in \Lambda , \\& W_{p} = \sum_{q=1}^{n} \bar{K}_{pq} \bigl(\bar{b}_{pq}^{R}+ \bar{b}_{pq}^{I}+\bar{b}_{pq}^{J} + \bar{b}_{pq}^{K} \bigr) \bigl(\beta _{q}^{R}+ \beta _{q}^{I}+\beta _{q}^{J}+\beta _{q}^{K} \bigr) ,\quad p\in \Lambda . \end{aligned}$$

### Theorem 1

*Suppose that* $(S_{1})$*–*$(S_{5})$ *hold*. *Then system* (8) *has a unique pseudo almost periodic solution in the region* $\mathbb{X}^{\ast }=\{\varphi \mid \varphi \in \mathbb{X}, \Vert \varphi \Vert _{\mathbb{X}}\leq \kappa \}$.

### Proof

Let $\varphi =(\varphi _{1}^{R},\varphi _{1}^{I},\varphi _{1}^{J},\varphi _{1}^{K},\dots ,\varphi _{n}^{R},\varphi _{n}^{I},\varphi _{n}^{J}, \varphi _{n}^{K})^{T}\in \mathbb{X}$. Obviously, $(S_{1})$ implies that $F_{q}[t,\varphi ]$ and $G_{q}[t,u,\varphi ]$ are uniformly continuous functions on $\mathbb{R}$ for $q\in \Lambda $. Set $h(t,z)=\varphi _{q}(t-z)$ $(q\in \Lambda )$, where $\varphi _{q}(t-z)=(\varphi _{q}^{R}(t-z),\varphi _{q}^{I}(t-z),\varphi _{q}^{J}(t-z),\varphi _{q}^{K}(t-z))$. By Theorem 5.3 in [40] and Definition 5.7 in [40], we can obtain that $h\in PAP(\mathbb{R}\times \Omega )$ and *h* is continuous in $z\in K$ and uniformly in $t\in \mathbb{R}$ for all compact subset *K* of $\Omega \subset \mathbb{R}$. This, together with $\tau _{pq}\in PAP(\mathbb{R},\mathbb{R}^{+})$ and Theorem 5.11 in [40], implies that

$$\begin{aligned} \varphi _{q} \bigl(t-\tau _{pq}(t) \bigr)\in PAP \bigl( \mathbb{R},\mathbb{R}^{4} \bigr),\quad p,q\in \Lambda . \end{aligned}$$

Again from Corollary 5.4 in [40], we have

$$\begin{aligned} F_{q}[t,\varphi ]\in PAP \bigl(\mathbb{R}^{4}, \mathbb{R}^{4\times 1} \bigr),\quad q\in \Lambda , \end{aligned}$$

which implies that

$$\begin{aligned} \sum_{q=1}^{n}A_{pq}(t)F_{q}[t, \varphi ]\in PAP \bigl(\mathbb{R},\mathbb{R}^{4\times 1} \bigr),\quad p,q\in \Lambda . \end{aligned}$$

By a similar argument as that in the proof of Lemma 2.3 in [13], one can obtain that

$$\begin{aligned} \sum_{q=1}^{n}B_{pq}(t) \int _{0}^{\infty }K_{pq}(u)G_{q} \bigl(\varphi _{q}(t-u) \bigr)\,\mathrm{d}u\in PAP \bigl(\mathbb{R}, \mathbb{R}^{4\times 1} \bigr),\quad p\in \Lambda . \end{aligned}$$

For any $\varphi \in \mathbb{X}$, consider the following linear system:

9 $$\begin{aligned} X_{p}'(t) =&-c_{p}(t)X_{p}(t)+ \sum_{q=1}^{n}A_{pq}(t)F_{q}[t, \varphi ] \\ &+\sum_{q=1}^{n}B_{pq}(t) \int _{0}^{\infty }K_{pq}(u)G_{q}[t,u, \varphi ]\,\mathrm{d}u+U_{p}(t),\quad p\in \Lambda . \end{aligned}$$

In view of Lemma 2, we can conclude that the linear system

10 $$\begin{aligned} X_{p}'(t)=-c_{p}(t)X_{p}(t), \quad p\in \Lambda \end{aligned}$$

admits an exponential dichotomy. Furthermore, by Lemma 1, we obtain that system (9) has exactly one pseudo periodic almost solution:

$$ X^{\varphi }= \bigl(X^{\varphi }_{1},X^{\varphi }_{2}, \ldots ,X^{\varphi }_{n} \bigr), $$

where

11 $$\begin{aligned} X_{p}^{\varphi }(t) =& \int _{-\infty }^{t}e^{-\int _{s}^{t}c_{p}(u)\,\mathrm{d}u} \Biggl( \sum _{q=1}^{n}A_{pq}(s)F_{q}[s, \varphi ] \\ &+\sum_{q=1}^{n}B_{pq}(s) \int _{0}^{\infty }K_{pq}(u)G_{q}[s,u, \varphi ]\,\mathrm{d}u+U_{p}(s) \Biggr) \,\mathrm{d}s,\quad p\in \Lambda . \end{aligned}$$

Define a mapping $T: \mathbb{X}\rightarrow \mathbb{X}$ by setting $(T\varphi )(t)=X^{\varphi }(t)$, $\forall \,\varphi \in \mathbb{X}$. Obviously, $\mathbb{X}^{\ast }$ is a closed convex subset of $\mathbb{X}$.

Now, we prove that the mapping *T* is a self-mapping from $\mathbb{X}^{\ast }$ to $\mathbb{X}^{\ast }$. In fact, for $\forall \,\varphi \in \mathbb{X}^{\ast }$, we have

12 $$\begin{aligned} &\sup_{t\in \mathbb{R}}\bigl\vert (T\varphi )_{p}^{R}(t) \bigr\vert \\ &\quad =\sup_{t\in \mathbb{R}} \Biggl\vert \int _{-\infty }^{t}e^{-\int _{s}^{t}c_{p}(u)\,\mathrm{d}u} \Biggl( \sum _{q=1}^{n} \bigl(a_{pq}^{R}(s)f_{q}^{R}[s, \phi ] -a_{pq}^{I}(s)f_{q}^{I}[s,\phi ] \\ &\quad \quad {}-a_{pq}^{J}(s)f_{q}^{J}[s,\phi ]-a_{pq}^{K}(s)f_{q}^{K}[s,\phi ] \bigr) +\sum_{q=1}^{n} \biggl(b_{pq}^{R}(s) \int _{0}^{\infty }K_{pq}(u) \tilde{g}_{q}^{R}\,\mathrm{d}u \\ &\quad \quad {} -b_{pq}^{I}(s) \int _{0}^{\infty }K_{pq}(u) \tilde{g}_{q}^{I}\,\mathrm{d}u -b_{pq}^{J}(s) \int _{0}^{\infty }K_{pq}(u) \tilde{g}_{q}^{J}\,\mathrm{d}u \\ &\quad \quad {} -b_{pq}^{K}(s) \int _{0}^{\infty }K_{pq}(u) \tilde{g}_{q}^{K}\,\mathrm{d}u \biggr)+u_{p}^{R}(s) \Biggr) \,\mathrm{d}s \Biggr\vert \\ &\quad \leq \sup_{t\in \mathbb{R}} \int _{-\infty }^{t}e^{-\int _{s}^{t}c_{p}(u)\,\mathrm{d}u} \Biggl( \sum _{q=1}^{n} \bigl(\bar{a}_{pq}^{R} +\bar{a}_{pq}^{I}+\bar{a}_{pq}^{J}+ \bar{a}_{pq}^{K} \bigr) \\ &\quad \quad {}\times \bigl(\alpha _{q}^{R} \bigl\vert \varphi_{q}^{R} \bigl(s-\tau _{pq}(s) \bigr) \bigr\vert +\alpha _{q}^{I} \bigl\vert \varphi _{q}^{I} \bigl(s-\tau_{pq}(s) \bigr) \bigr\vert +\alpha _{q}^{J} \bigl\vert \varphi_{q}^{J} \bigl(s-\tau _{pq}(s) \bigr) \bigr\vert \\ &\quad \quad {} +\alpha _{q}^{K} \bigl\vert \varphi _{q}^{K} \bigl(s-\tau _{pq}(s) \bigr) \bigr\vert \bigr) +\sum_{q=1}^{n} \bigl( \bar{b}_{pq}^{R}+\bar{b}_{pq}^{I}+ \bar{b}_{pq}^{J} +\bar{b}_{pq}^{K} \bigr) \\ &\quad \quad {}\times \int _{0}^{\infty } \bigl\vert K_{pq}(u) \bigr\vert \bigl(\beta _{q}^{R} \bigl\vert \varphi _{q}^{R}(s-u) \bigr\vert + \beta _{q}^{I} \bigl\vert \varphi _{q}^{I}(s-u) \bigr\vert + \beta _{q}^{J} \bigl\vert \varphi _{q}^{J}(s-u) \bigr\vert \\ &\quad \quad {} +\beta _{q}^{K} \bigl\vert \varphi _{q}^{K}(s-u) \bigr\vert \bigr) \,\mathrm{d}u+ \bar{u}_{p}^{R} \Biggr)\,\mathrm{d}s \\ &\quad \leq \sup_{t\in \mathbb{R}} \int _{-\infty }^{t}e^{-\int _{s}^{t}c_{p}(u)\,\mathrm{d}u} \Biggl( \sum _{q=1}^{n} \bigl(\bar{a}_{pq}^{R} +\bar{a}_{pq}^{I}+\bar{a}_{pq}^{J}+ \bar{a}_{pq}^{K} \bigr) \bigl(\alpha _{q}^{R}+ \alpha _{q}^{I}+\alpha _{q}^{J} \\ &\quad \quad {}+\alpha _{q}^{K} \bigr)\Vert \varphi \Vert _{\mathbb{X}} +\sum_{q=1}^{n} \bar{K}_{pq} \bigl( \bar{b}_{pq}^{R}+ \bar{b}_{pq}^{I}+ \bar{b}_{pq}^{J} + \bar{b}_{pq}^{K} \bigr) \bigl(\beta _{q}^{R}+ \beta _{q}^{I}+ \beta _{q}^{J} \\ &\quad \quad {}+\beta _{q}^{K} \bigr)\Vert \varphi \Vert _{\mathbb{X}} + \bar{u}_{p}^{R} \Biggr)\,\mathrm{d}s \\ &\quad \leq \frac{1}{\underline{c}_{p}} \bigl(V_{p}\kappa +W_{p}\kappa + \bar{u}_{p}^{R} \bigr) =\frac{\Theta _{p}\kappa +\bar{u}_{p}^{R}}{\underline{c}_{p}},\quad p\in \Lambda . \end{aligned}$$

In a similar way, we can obtain

13 $$\begin{aligned} \sup_{t\in \mathbb{R}}\bigl\vert (T\varphi )_{p}(t) \bigr\vert \leq \frac{\Theta _{p}\kappa +\bar{u}_{p}^{l}}{\underline{c}_{p}},\quad p\in \Lambda ,l=I,J,K. \end{aligned}$$

It follows from (12), (13), and $(H_{4})$ that

$$\begin{aligned} \Vert T\varphi \Vert _{\mathbb{X}}\leq \kappa , \end{aligned}$$

which implies that $T\varphi \in \mathbb{X}^{\ast }$. Therefore, the mapping *T* is a self-mapping from $\mathbb{X}^{\ast }$ to $\mathbb{X}^{\ast }$. Next, we show that $T:\mathbb{X}^{\ast }\rightarrow \mathbb{X}^{\ast }$ is a contraction mapping. In fact, for any $\varphi , \psi \in \mathbb{X}^{\ast }$, we have

14 $$\begin{aligned} &\sup_{t\in \mathbb{R}}\bigl\vert (T\varphi )_{p}^{R}(t)-(T\psi )_{p}^{R}(t) \bigr\vert \\ &\quad \leq \sup_{t\in \mathbb{R}}\int _{-\infty }^{t}e^{-\int _{s}^{t}c_{p}(u)\,\mathrm{d}u} \Biggl(\sum _{q=1}^{n} \bigl(\bar{a}_{pq}^{R}+ \bar{a}_{pq}^{I}+\bar{a}_{pq}^{J}+ \bar{a}_{pq}^{K} \bigr) \bigl(\alpha _{q}^{R} \bigl\vert \varphi _{q}^{R} \bigl(s-\tau _{pq}(s) \bigr) \\ &\quad \quad {}-\psi _{q}^{R} \bigl(s-\tau _{pq}(s) \bigr) \bigr\vert + \alpha _{q}^{I} \bigl\vert \varphi _{q}^{I} \bigl(s-\tau _{pq}(s) \bigr)-\psi _{q}^{I} \bigl(s-\tau _{pq}(s) \bigr) \bigr\vert \\ &\quad \quad {} +\alpha _{q}^{J} \bigl\vert \varphi _{q}^{J} \bigl(s-\tau _{pq}(s) \bigr)-\psi _{q}^{J} \bigl(s-\tau _{pq}(s) \bigr) \bigr\vert +\alpha _{q}^{K} \bigl\vert \varphi _{q}^{K} \bigl(s-\tau _{pq}(s) \bigr) \\ &\quad \quad {}-\psi _{q}^{K} \bigl(s-\tau _{pq}(s) \bigr) \bigr\vert \bigr) +\sum_{q=1}^{n} \bigl( \bar{b}_{pq}^{R}+\bar{b}_{pq}^{I}+ \bar{b}_{pq}^{J} +\bar{b}_{pq}^{K} \bigr) \int _{0}^{\infty } \bigl\vert K_{pq}(u) \bigr\vert \\ &\quad \quad {}\times \bigl(\beta _{q}^{R} \bigl\vert \varphi _{q}^{R}(s-u)-\psi _{q}^{R}(s-u) \bigr\vert +\beta _{q}^{I} \bigl\vert \varphi _{q}^{I}(s-u)-\psi _{q}^{I}(s-u) \bigr\vert \\ &\quad \quad {}+\beta _{q}^{J} \bigl\vert \varphi _{q}^{J}(s-u)- \psi _{q}^{J}(s-u) \bigr\vert +\beta _{q}^{K} \bigl\vert \varphi _{q}^{K}(s-u)-\psi _{q}^{K}(s-u) \bigr\vert \bigr) \,\mathrm{d}u \Biggr)\,\mathrm{d}s \\ &\quad \leq \sup_{t\in \mathbb{R}} \int _{-\infty }^{t}e^{-\int _{s}^{t}c_{p}(u)\,\mathrm{d}u} \Biggl(\sum _{q=1}^{n} \bigl(\bar{a}_{pq}^{R} +\bar{a}_{pq}^{I}+\bar{a}_{pq}^{J}+ \bar{a}_{pq}^{K} \bigr) \bigl(\alpha _{q}^{R}+ \alpha _{q}^{I} \\ &\quad \quad {}+\alpha _{q}^{J}+\alpha _{q}^{K} \bigr)\Vert \varphi -\psi \Vert _{\mathbb{X}}+\sum _{q=1}^{n} \bar{K}_{pq} \bigl( \bar{b}_{pq}^{R} + \bar{b}_{pq}^{I}+ \bar{b}_{pq}^{J} + \bar{b}_{pq}^{K} \bigr) \bigl(\beta _{q}^{R}+ \beta _{q}^{I} \\ &\quad \quad {}+\beta _{q}^{J} +\beta _{q}^{K} \bigr)\Vert \varphi -\psi \Vert _{\mathbb{X}} \Biggr)\,\mathrm{d}s \\ &\quad \leq \frac{1}{\underline{c}_{p}} (V_{p}+W_{p} )\Vert \varphi - \psi \Vert _{\mathbb{X}} =\frac{\Theta _{p}}{\underline{c}_{p}}\Vert \varphi -\psi \Vert _{\mathbb{X}}. \end{aligned}$$

In a similar way, we can obtain

15 $$\begin{aligned} \sup_{t\in \mathbb{R}}\bigl\vert (T\varphi )_{p}^{l}(t)-(T\psi )_{p}^{l}(t) \bigr\vert \leq \frac{\Theta _{p}}{\underline{c}_{p}}\Vert \varphi -\psi \Vert _{\mathbb{X}}, \quad p\in \Lambda , l=I,J,K. \end{aligned}$$

It follows from (14), (15), and $(H_{4})$ that

$$\begin{aligned} \bigl\Vert T(\varphi )-T(\psi ) \bigr\Vert _{\mathbb{X}}\leq \rho \Vert \varphi -\psi \Vert _{\mathbb{X}}. \end{aligned}$$

Hence, *T* is a contraction mapping from $\mathbb{X}^{\ast }$ to $\mathbb{X}^{\ast }$. Therefore, *T* has a unique fixed point in $\mathbb{X}^{\ast }$, that is, (8) has a unique pseudo almost periodic solution in $\mathbb{X}^{\ast }$. The proof is complete. □

By Remark 1, Theorem 1, we have the following.

### Theorem 2

*Suppose that* $(S_{1})$*–*$(S_{5})$ *hold*, *then system* (1) *has a unique pseudo almost periodic solution in* $\mathbb{X}^{\ast }=\{\varphi \mid \varphi \in \mathbb{X}, \Vert \varphi \Vert _{\mathbb{X}}\leq \kappa \}$.

### Definition 5

Let $x=(x_{1}^{R},x_{1}^{I},x_{1}^{J},x_{1}^{K},\dots ,x_{n}^{R},x_{n}^{I},x_{n}^{J},x_{n}^{K})^{T}$ be a solution of (8) with the initial value $\varphi =(\varphi _{1}^{R},\varphi _{1}^{I},\varphi _{1}^{J},\varphi _{1}^{K},\dots ,\varphi _{n}^{R},\varphi _{n}^{I}, \varphi _{n}^{J}, \varphi _{n}^{K})^{T}\in C((-\infty ,0],\mathbb{R}^{4n})$ and $y=(y_{1}^{R},y_{1}^{I},y_{1}^{J}, y_{1}^{K},\dots ,y_{n}^{R}, y_{n}^{I},y_{n}^{J},y_{n}^{K})^{T}$ be an arbitrary solution of system (8) with the initial value $\psi =(\psi _{1}^{R},\psi _{1}^{I},\psi _{1}^{J},\psi _{1}^{K}, \dots ,\psi _{n}^{R},\psi _{n}^{I},\psi _{n}^{J},\psi _{n}^{K})^{T}\in C((-\infty ,0],\mathbb{R}^{4n})$. If there exist constants $\lambda >0$ and $M>0$ such that

$$ \bigl\Vert x(t)-y(t) \bigr\Vert \leq M\Vert \varphi -\psi \Vert e^{-\lambda t}, \quad t>0, $$

where

$$ \bigl\Vert x(t)-y(t) \bigr\Vert =\max_{p\in \Lambda } \bigl\{ \bigl\vert x_{p}^{l}(t)-y_{p}^{l}(t) \bigr\vert , l\in E \bigr\} , $$

$$ \Vert \varphi -\psi \Vert _{0}=\max_{p\in \Lambda , l\in E} \Bigl\{ \sup_{s\in (-\infty ,0]}\bigl\vert \varphi _{p}^{l}(s)- \psi_{p}^{l}(s) \bigr\vert \Bigr\} . $$

Then the solution *x* of system (8) is said to be globally exponentially stable.

### Theorem 3

*Under the assumptions of Theorem *1, *system* (8) *has a unique pseudo almost periodic solution that is globally exponentially stable*.

### Proof

From Theorem 1, we see that system (8) has a pseudo almost periodic solution $X^{\ast }=(X_{1}^{\ast },X_{2}^{\ast },\ldots ,X_{n}^{\ast })^{T}$ with initial value $\Phi ^{\ast }=(\phi _{1}^{\ast },\phi _{2}^{\ast },\ldots ,\phi _{n}^{\ast })^{T}$. Suppose that $X=(X_{1},X_{2},\ldots ,X_{n})^{T}$ is an arbitrary solution of system (8) with initial value $\Phi =(\phi _{1},\phi _{2},\ldots ,\phi _{n})^{T}$ and let $Z=X-X^{\ast }$, then we have

16 $$\begin{aligned} Z_{p}'(t) =&-c_{p}(t)Z_{p}(t)+ \sum_{q=1}^{n}A_{pq}(t) \bigl(F_{q} \bigl[t,z+x^{*} \bigr]-F_{q} \bigl[t,x^{*} \bigr] \bigr) \\ &+\sum_{q=1}^{n}B_{pq}(t) \int _{0}^{\infty }K_{pq}(u) \bigl(G_{q} \bigl[t,u,z+x^{*} \bigr] \\ &{} -G_{q} \bigl[t,u,x^{*} \bigr] \bigr)\,\mathrm{d}u,\quad p\in \Lambda . \end{aligned}$$

The initial condition of (16) is

$$\begin{aligned} Z_{p}(s)=\psi _{p}(s)=\Phi _{p}(s)-\Phi _{p}^{\ast }(s), \quad s\in (-\infty ,0], p\in \Lambda . \end{aligned}$$

For $p\in \Lambda $, we define $\Gamma _{p}$ as follows:

$$\begin{aligned} \Gamma _{p}(\theta )=\theta -\underline{c}_{p} +V_{p}e^{\theta \bar{\tau }_{pq}}+W_{p},\quad p\in \Lambda . \end{aligned}$$

From $(S_{5})$, we have

$$\begin{aligned} \Gamma _{p}(0)=-\underline{c}_{p} +V_{p}+W_{p}=-\underline{c}_{p}+\Theta _{p}< 0 \end{aligned}$$

and $\Gamma _{p}(\theta )$ is continuous on $[0,+\infty )$ and $\Gamma _{p}(\theta )\rightarrow +\infty $, as $\theta \rightarrow +\infty $. Hence, there exists $\xi _{p}> 0$ such that $\Gamma _{p}(\xi _{p})=0$ and $\Gamma _{p}(\theta )<0$ for $\theta \in (0,\xi _{p})$, $p\in \Lambda $. So, we can choose a positive constant $0< \lambda < \min \{\min_{p\in \Lambda }\xi _{p},\min_{p\in \Lambda }\{\underline{c}_{p}\} \}$ such that

$$\begin{aligned} \Gamma _{p}(\lambda )< 0,\quad p\in \Lambda . \end{aligned}$$

Let $\gamma _{p}=V_{p}e^{\lambda \bar{\tau }_{pq}}+W_{p}$, $p\in \Lambda $. Then $\gamma _{p}<\underline{c}_{p}-\lambda $, $p\in \Lambda $. Take a constant *M* such that

$$\begin{aligned} M>\frac{\underline{c}_{p}-\lambda }{\gamma _{p}}>1,\quad p\in \Lambda , \end{aligned}$$

which yields

$$\begin{aligned} \frac{1}{M}-\frac{\gamma _{p}}{\underline{c}_{p}-\lambda }< 0,\quad p\in \Lambda . \end{aligned}$$

Hence, for any $\epsilon >0$, it is obvious that

17 $$\begin{aligned} \bigl\Vert Z(0) \bigr\Vert < \bigl(\Vert \varphi \Vert _{0}+\epsilon \bigr) \end{aligned}$$

and

18 $$\begin{aligned} \bigl\Vert Z(t) \bigr\Vert < \bigl(\Vert \varphi \Vert _{0}+\epsilon \bigr)e^{-\lambda t}< M\bigl(\Vert \varphi \Vert _{0}+\epsilon \bigr)e^{-\lambda t},\quad \forall \, t\in (- \infty ,0]. \end{aligned}$$

We claim that

19 $$\begin{aligned} \bigl\Vert Z(t) \bigr\Vert < M\bigl(\Vert \varphi \Vert _{0}+\epsilon \bigr)e^{-\lambda t},\quad \forall \,t>0. \end{aligned}$$

Otherwise, there must exist some $p\in \Lambda $ and $\eta >0$ such that

20 $$\begin{aligned} \textstyle\begin{cases} \vert Z_{p}(\eta ) \vert =\Vert Z(\eta ) \Vert =M(\Vert \varphi \Vert _{0}+\epsilon )e^{-\lambda \eta }, \\ \Vert Z(t) \Vert < M(\Vert \varphi \Vert _{0}+\epsilon )e^{-\lambda t},\quad t< \eta . \end{cases}\displaystyle \end{aligned}$$

Multiplying both sides of (16) by $e^{\int _{0}^{t}c_{p}(u)\,\mathrm{d}u}$ and integrating over $[0,t]$, we get

$$\begin{aligned} Z_{p}(t) =&Z_{p}(0)e^{-\int _{0}^{t}c_{p}(u)\,\mathrm{d}u} + \int _{0}^{t}e^{-\int _{s}^{t}c_{p}(u)\,\mathrm{d}u} \Biggl(\sum _{q=1}^{n}A_{pq}(s) \bigl(F_{q} \bigl[s,z+x^{*} \bigr] \\ &{} -F_{q} \bigl[s,x^{*} \bigr] \bigr) +\sum _{q=1}^{n}B_{pq}(s) \int _{0}^{\infty }K_{pq}(u) \bigl(G_{q} \bigl[s,u,z+x^{*} \bigr] \\ &{} -G_{q} \bigl[s,u,x^{*} \bigr] \bigr)\,\mathrm{d}u \Biggr) \,\mathrm{d}s. \end{aligned}$$

From this and (20), we get

21 $$\begin{aligned} &\bigl\vert z_{p}^{R}(\eta ) \bigr\vert \\ &\quad =\Biggl\vert z_{p}^{R}(0)e^{-\int _{0}^{\eta }c_{p}(u)\,\mathrm{d}u} + \int _{0}^{\eta }e^{-\int _{s}^{\eta }c_{p}(u)\,\mathrm{d}u} \Biggl(\sum _{q=1}^{n} \bigl(a_{pq}^{R}(s) \hat{f}_{q}^{R} \bigl[s,x-x^{*} \bigr] \\ &\quad \quad {} -a_{pq}^{I}(s)\hat{f}_{q}^{I} \bigl[s,x-x^{*} \bigr]-a_{pq}^{J}(s) \hat{f}_{q}^{J} \bigl[s,x-x^{*} \bigr]-a_{pq}^{K}(s)\hat{f}_{q}^{K} \bigl[s,x-x^{*} \bigr] \bigr) \\ &\quad \quad {} +\sum_{q=1}^{n} \biggl(b_{pq}^{R}(s) \int _{0}^{\infty }K_{pq}(u) \hat{g}_{q}^{R} \bigl[s,u,x-x^{*} \bigr] -b_{pq}^{I}(s) \int _{0}^{\infty }K_{pq}(u) \\ &\quad \quad {}\times \hat{g}_{q}^{I} \bigl[s,u,x-x^{*} \bigr]-b_{pq}^{J}(s) \int _{0}^{\infty }K_{pq}(u) \hat{g}_{q}^{J} \bigl[s,u,x-x^{*} \bigr] \\ &\quad \quad {} -b_{pq}^{K}(s) \int _{0}^{\infty }K_{pq}(u) \hat{g}_{q}^{K} \bigl[s,u,x-x^{*} \bigr] \biggr) \,\mathrm{d}u \Biggr)\,\mathrm{d}s \Biggr\vert \\ &\quad \leq \bigl(\Vert \varphi \Vert _{0}+\epsilon \bigr)e^{-\int _{0}^{\eta }c_{p}(u)\,\mathrm{d}u} + \int _{0}^{\eta }e^{-\int _{s}^{\eta }c_{p}(u)\,\mathrm{d}u} \Biggl(\sum _{q=1}^{n} \bigl(\bar{a}_{pq}^{R}+ \bar{a}_{pq}^{I} \\ &\quad \quad {}+\bar{a}_{pq}^{J}+\bar{a}_{pq}^{K} \bigr) \bigl(\alpha _{q}^{R}+\alpha _{q}^{I}+ \alpha _{q}^{J}+\alpha _{q}^{K} \bigr)e^{\lambda \bar{\tau }_{pq}} +\sum_{q=1}^{n} \bigl( \bar{b}_{pq}^{R}+\bar{b}_{pq}^{I} \\ &\quad \quad {}+\bar{b}_{pq}^{J} +\bar{b}_{pq}^{K} \bigr) \bigl(\beta _{q}^{R}+\beta _{q}^{I}+ \beta _{q}^{J}+\beta _{q}^{K} \bigr) \int _{0}^{\infty } \bigl\vert K_{pq}(u) \bigr\vert e^{-\lambda s}e^{\lambda u}\,\mathrm{d}u \Biggr)\,\mathrm{d}s \\ &\quad \quad {}\times M\bigl(\Vert \varphi \Vert _{0}+\epsilon \bigr) \\ &\quad \leq \bigl(\Vert \varphi \Vert _{0}+\epsilon \bigr)e^{-\lambda \eta }e^{-\int _{0}^{\eta }(c_{p}(u)-\lambda )\,\mathrm{d}u} + \int _{0}^{\eta }e^{-\int _{s}^{\eta }c_{p}(u)\,\mathrm{d}u} \Biggl(\sum _{q=1}^{n} \bigl(\bar{a}_{pq}^{R} \\ &\quad \quad {}+\bar{a}_{pq}^{I}+\bar{a}_{pq}^{J}+ \bar{a}_{pq}^{K} \bigr) \bigl(\alpha _{q}^{R}+ \alpha _{q}^{I}+\alpha _{q}^{J}+\alpha _{q}^{K} \bigr) e^{\lambda \bar{\tau }_{pq}} +\sum _{q=1}^{n}\bar{K}_{pq} \bigl( \bar{b}_{pq}^{R} \\ &\quad \quad {}+\bar{b}_{pq}^{I}+\bar{b}_{pq}^{J} + \bar{b}_{pq}^{K} \bigr) \bigl(\beta _{q}^{R}+ \beta _{q}^{I}+\beta _{q}^{J}+\beta _{q}^{K} \bigr) \Biggr)\,\mathrm{d}s M\bigl(\Vert \varphi \Vert _{0}+\epsilon \bigr)e^{-\lambda \eta } \\ &\quad \leq \bigl(\Vert \varphi \Vert _{0}+\epsilon \bigr)e^{-\lambda \eta }e^{-\int _{0}^{\eta }(c_{p}(u)-\lambda )\,\mathrm{d}u} + \frac{1-e^{(\lambda -\underline{c}_{p})\eta }}{\underline{c}_{p}-\lambda } \bigl(V_{p}e^{\lambda \bar{\tau }_{pq}} \\ &\quad \quad {}+W_{p} \bigr) M\bigl(\Vert \varphi \Vert _{0}+ \epsilon \bigr)e^{-\lambda \eta } \\ &\quad \leq M\bigl(\Vert \varphi \Vert _{0}+\epsilon \bigr)e^{-\lambda \eta } \biggl[ \biggl( \frac{1}{M}-\frac{\gamma _{p}}{\underline{c}_{p}-\lambda } \biggr)e^{(\lambda -\underline{c}_{p})\eta } +\frac{\gamma _{p}}{\underline{c}_{p}-\lambda } \biggr] \\ &\quad < M\bigl(\Vert \varphi \Vert _{0}+\epsilon \bigr)e^{-\lambda \eta }, \end{aligned}$$

where $\hat{f}_{q}^{l}[s,x-x^{*}]\triangleq f_{q}^{l}[s,x]-f_{q}^{l}[s,x^{*}], \hat{g}_{q}^{l}[s,u,x-x^{*}] \triangleq g_{q}^{l}[s,u,x]-g_{q}^{l}[s,u,x^{*}]$.

Similarly, we can get

22 $$\begin{aligned} \bigl\vert z_{p}^{l}(\eta ) \bigr\vert < M\bigl(\Vert \varphi \Vert _{0}+\epsilon \bigr)e^{-\lambda \eta }, \quad l=I,J,K. \end{aligned}$$

It follows from (21) and (22) that

$$\begin{aligned} \bigl\vert Z_{p}(\eta ) \bigr\vert < M\bigl(\Vert \varphi \Vert _{0}+\epsilon \bigr)e^{-\lambda \eta }, \end{aligned}$$

which contradicts the first equation of (20). Hence, (19) holds. Letting $\epsilon \rightarrow 0^{+}$, from (19), we have

$$\begin{aligned} \bigl\Vert Z(t) \bigr\Vert \leq M\Vert \varphi \Vert _{0}e^{-\lambda t},\quad \forall \,t>0. \end{aligned}$$

Therefore, the pseudo almost periodic solution of system (8) is globally exponentially stable. The proof is complete. □

By Remark 1, Theorem 3, we have

### Theorem 4

*Suppose that* $(S_{1})$*–*$(S_{5})$ *hold*, *then system* (1) *has a unique pseudo almost periodic solution that is globally exponentially stable*.

## An example

In this section, we give an example to illustrate the feasibility and effectiveness of our results obtained in Sect. 3.

### Example 1

Consider the following quaternion-valued system:

23 $$\begin{aligned} x_{p}'(t) =&-c_{p}(t)x_{p}(t)+ \sum_{q=1}^{2}a_{pq}(t)f_{q} \bigl(x_{q} \bigl(t-\tau _{pq}(t) \bigr) \bigr) \\ &+\sum_{q=1}^{2}b_{pq}(t) \int _{0}^{\infty }K_{pq}(u)g_{q} \bigl(x_{q}(t-u) \bigr)du+u_{p}(t), \end{aligned}$$

where $p=1,2$, $x_{p}=x_{p}^{R}+ix_{p}^{I}+jx_{p}^{J}+kx_{p}^{K}\in \mathbb{Q}$, and the coefficients are taken as follows:

$$\begin{aligned}& c_{1}(t)=4+\cos (\sqrt{2}t),\qquad c_{2}(t)=5-\sin t,\qquad K_{pq}(u)=\vert \sin u \vert e^{-4u}, \\& f_{q}(x_{q})=\frac{1}{4}\sin ^{2} \bigl(x_{q}^{R}+x_{q}^{J} \bigr)+i\bigl\vert x_{q}^{I}+x_{q}^{K} \bigr\vert +j \frac{1}{2}\sin x_{q}^{J}+k\frac{1}{2} \bigl(\bigl\vert x_{q}^{K}+1 \bigr\vert +\bigl\vert x_{q}^{K} \bigr\vert -1 \bigr), \\& g_{q}(x_{q})=\tan x_{q}^{R}+i \frac{1}{8}\sin ^{2} \bigl(x_{q}^{R}+x_{q}^{I} \bigr)+j\bigl\vert x_{q}^{J} \bigr\vert +k \frac{1}{4}\sin \bigl(\sqrt{2} x_{q}^{K} \bigr), \\& a_{11}(t)=a_{12}(t)=0.032\cos t+i 0.03\sin ( \sqrt{2}t)+j0.028 \sin t+k0.045\cos t, \\& a_{21}(t)=a_{22}(t)=0.04\sin (\sqrt{2} t)+i 0.05\sin ( \sqrt{3} t)+j0.036\cos (\sqrt{2} t)+k0.06\sin t, \\& b_{11}(t)=b_{12}(t)=0.4\sin t+i 0.2\cos (\sqrt{2} t)+j0.3 \sin (\sqrt{3} t)+k0.25\cos t, \\& b_{21}(t)=b_{22}(t)=0.5\cos (\sqrt{3} t)+i 0.45\sin t+j0.35 \cos t+k0.6\sin (\sqrt{2} t), \\& \tau _{11}(t)=\bigl\vert \sin (2t) \bigr\vert ,\qquad \tau _{12}(t)=\cos ^{2}t,\qquad \tau _{21}(t)=\bigl\vert \sin (\sqrt{2}t) \bigr\vert , \qquad \tau _{22}(t)=\sin ^{2}t, \\& u_{1}(t)=u_{2}(t)=\cos t+i\sin (2t)+j\sin (\sqrt{2} t)+k \cos (\sqrt{2}t). \end{aligned}$$

By a simple calculation, we have

$$\begin{aligned}& \underline{c}_{1}=3,\qquad \underline{c}_{2}=4, \qquad \bar{K}_{pq}=\frac{1}{4},\qquad p,q=1,2, \\& \alpha _{q}^{R}=\alpha _{q}^{J}= \frac{1}{2},\qquad \alpha _{q}^{I}=\alpha _{q}^{K}=\beta _{q}^{R}=\beta _{q}^{J}=1,\qquad \beta _{q}^{I}=\beta _{q}^{K}=\frac{1}{4}, \\& \bar{a}_{11}^{R}=\bar{a}_{12}^{R}=0.032, \qquad \bar{a}_{11}^{I}=\bar{a}_{12}^{I}=0.03, \qquad \bar{a}_{11}^{J}=\bar{a}_{12}^{J}=0.028, \\& \bar{a}_{11}^{K}=\bar{a}_{12}^{K}=0.045, \qquad \bar{a}_{21}^{R}=\bar{a}_{22}^{R}=0.04, \qquad \bar{a}_{21}^{I}=\bar{a}_{22}^{I}=0.05, \\& \bar{a}_{21}^{J}=\bar{a}_{22}^{J}=0.036, \qquad \bar{a}_{21}^{K}=\bar{a}_{22}^{K}=0.06, \qquad \bar{b}_{11}^{R}=\bar{b}_{12}^{R}=0.4, \\& \bar{b}_{11}^{I}=\bar{b}_{12}^{I}=0.2, \qquad \bar{b}_{11}^{J}=\bar{b}_{12}^{J}=0.3, \qquad \bar{b}_{11}^{K}=\bar{b}_{12}^{K}=0.25, \\& \bar{b}_{21}^{R}=\bar{b}_{22}^{R}=0.5, \qquad \bar{b}_{21}^{I}=\bar{b}_{22}^{I}=0.45, \qquad \bar{b}_{21}^{J}=\bar{b}_{22}^{J}=0.35, \qquad \bar{b}_{21}^{K}=\bar{b}_{22}^{K}=0.6, \\& \bar{u}_{1}^{R}=\bar{u}_{1}^{I}= \bar{u}_{1}^{J}=\bar{u}_{1}^{K}= \bar{u}_{2}^{R} =\bar{u}_{2}^{I}= \bar{u}_{2}^{J}=\bar{u}_{2}^{K}=1,\qquad \bar{\tau }_{pq}=1,\qquad p,q=1,2. \end{aligned}$$

Take $\kappa =2$, then we have

$$\begin{aligned} &\max \biggl\{ \frac{2\Theta _{1}+\bar{u}_{1}^{R}}{\underline{c}_{1}},\, \frac{2\Theta _{1}+\bar{u}_{1}^{I}}{\underline{c}_{1}},\, \frac{2\Theta _{1}+\bar{u}_{1}^{J}}{\underline{c}_{1}},\, \frac{2\Theta _{1}+\bar{u}_{1}^{K}}{\underline{c}_{1}},\, \frac{2\Theta _{2}+\bar{u}_{2}^{R}}{\underline{c}_{2}},\, \frac{2\Theta _{2}+\bar{u}_{2}^{I}}{\underline{c}_{2}}, \\ &\qquad \frac{2\Theta _{2}+\bar{u}_{2}^{J}}{\underline{c}_{2}},\, \frac{2\Theta _{2}+\bar{u}_{2}^{K}}{\underline{c}_{2}} \biggr\} \approx \{1.8327, 1.9955\}=1.9955\leq \kappa =2 \end{aligned}$$

and

$$\begin{aligned} \max \biggl\{ \frac{\Theta _{1}}{\underline{c}_{1}},\quad \frac{\Theta _{2}}{\underline{c}_{2}} \biggr\} \approx \{0.7492, 0.8728\}= 0.8728=\rho < 1. \end{aligned}$$

**Figure 1:**
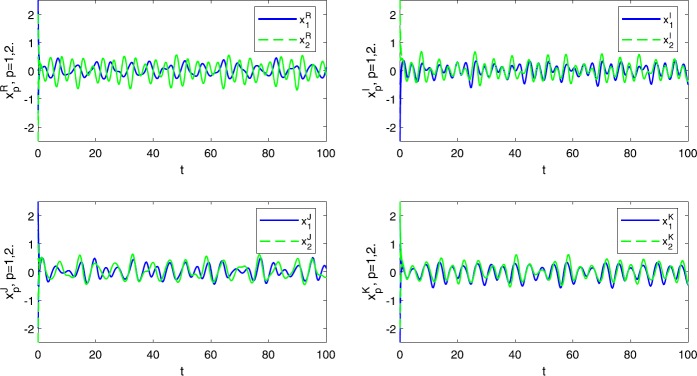
Transient states of four parts of QVNN (23) in Example 1

It is easy to check that all the assumptions in Theorem 4 are satisfied. Therefore, we obtain that (23) has a pseudo almost periodic solution that is globally exponentially stable (see Fig. 1).

### Remark 2

The results obtained in [13–15, 21, 27–34] cannot be applied to obtain that system (23) has a unique pseudo almost periodic solution that is globally exponentially stable.

## Conclusion

In this paper, we have established the existence and global exponential stability of pseudo almost periodic solutions of QVCNNs with discrete and distributed delays. An example has been given to demonstrate the effectiveness of our results. This is the first time to study the pseudo almost periodic oscillation for QVCNNs with discrete and distributed delays. Furthermore, the method of this paper can be used to study other types of quaternion-valued neural networks.
